# The Role of Mammalian Target of Rapamycin (mTOR) in Insulin Signaling

**DOI:** 10.3390/nu9111176

**Published:** 2017-10-27

**Authors:** Mee-Sup Yoon

**Affiliations:** Department of Molecular Medicine, School of Medicine, Gachon University, Incheon 21999, Korea; msyoon@gachon.ac.kr; Tel.: +82-32-899-6067

**Keywords:** insulin, mammalian target of rapamycin (mTOR), mTOR complex1 (mTORC1), mTOR complex2 (mTORC2), protein kinase B (PKB/Akt)

## Abstract

The mammalian target of rapamycin (mTOR) is a serine/threonine kinase that controls a wide spectrum of cellular processes, including cell growth, differentiation, and metabolism. mTOR forms two distinct multiprotein complexes known as mTOR complex 1 (mTORC1) and mTOR complex 2 (mTORC2), which are characterized by the presence of raptor and rictor, respectively. mTOR controls insulin signaling by regulating several downstream components such as growth factor receptor-bound protein 10 (Grb10), insulin receptor substrate (IRS-1), F-box/WD repeat-containing protein 8 (Fbw8), and insulin like growth factor 1 receptor/insulin receptor (IGF-IR/IR). In addition, mTORC1 and mTORC2 regulate each other through a feedback loop to control cell growth. This review outlines the current understanding of mTOR regulation in insulin signaling in the context of whole body metabolism.

## 1. Introduction

**Figure 1:**
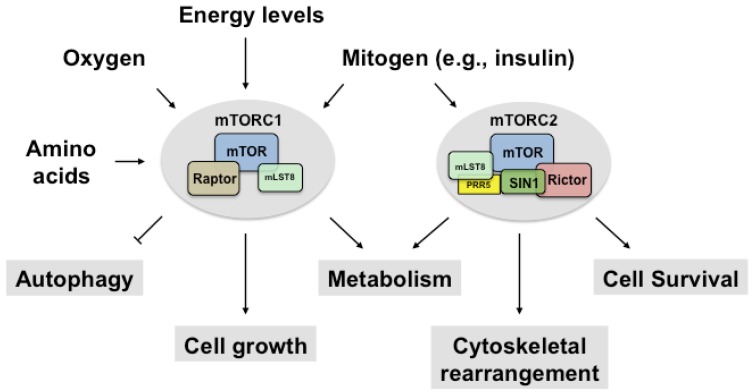
mTORC1 and mTORC2. mTOR is a serine/threonine kinase that forms two biochemically and functionally distinct complexes. mTORC1 consists of mTOR, raptor, mLST8 and the two inhibitory subunits, PRAS40 and DEPTOR, whereas mTORC2 consists of mTOR, rictor, mLST8, PRR5, SIN1 and the inhibitory subunit, DEPTOR. mTORC1 senses mitogens, oxygen levels, intracellular energy status, and amino acids to promote cell growth by regulating anabolic and catabolic processes. mTORC2 is activated by mitogen and controls cell survival, metabolism, and cytoskeletal organization. The inhibitory subunits in mTOR complexes are not presented here (PRAS40, DEPTOR). mTOR; mammalian target of rapamycin; mTORC1, mTOR complex 1; mTORC2, mTOR complex 2; mLST8, mammalian lethal with Sec13 protein 8; PRR5, proline-rich protein 5; SIN1, stress-activated map kinase-interacting protein 1; PRAS40, proline-rich Akt substrate of 40 kDa; DEPTOR, DEP domain-containing mTOR-interacting protein.

The mammalian target of rapamycin (mTOR) is a highly conserved serine/threonine (Ser/Thr) kinase that is involved in a diverse array of physiological processes, including cell metabolism, cell survival, cell growth, and autophagy [1]. In mammals, mTOR exists in two functionally distinct complexes, mTOR complex1 (mTORC1) and mTOR complex 2 (mTORC2) [2] (Figure 1).

mTORC1 consists of mTOR, regulatory-associated protein of mTOR (raptor), mammalian lethal with Sec13 protein 8 (mLST8), and the two inhibitory subunits, proline-rich Akt substrate of 40 kDa (PRAS40) and DEP domain-containing mTOR-interacting protein (DEPTOR) [1]. Rapamycin forms a complex with the peptidyl-prolyl-isomerase FKBP12 (12 kDa FK506-binding protein) and then binds to mTORC1 in a highly specific manner. However, it does not bind to mTORC2 [3]. mTORC2 is unresponsive to FKBP12-rapamycin [4,5], but prolonged treatment with rapamycin inhibits mTORC2 indirectly by preventing either de novo mTORC2 assembly or the synthesis of new mTOR via its inhibition [5,6]. mTORC2 contains mTOR, the rapamycin insensitive companion of the mammalian target of rapamycin (rictor), stress-activated map kinase (SAPK)-interacting 1 (SIN1), mLST8, proline-rich protein 5 (PRR5), proline-rich protein 5-like (PRR5L) (also known as protor1 and protor2) and DEPTOR. Whereas mTORC1 senses and integrates several extracellular and intracellular signals, including growth factors [7], the stimulus of mTORC2 is poorly understood; only growth factors are known to activate mTORC2 kinase activity [8].

The growth factor insulin is known as the most potent physiological anabolic agent [9]. Insulin is synthesized in pancreatic β cells of Langerhans islets as preproinsulin. It is then processed to proinsulin, converted to insulin and C-peptide, and stored in secretory granules awaiting release on demand [10]. Monomeric insulin consists of 21 amino acid residues in the “A” chain and 30 amino acid residues in the “B” chain bound by disulfide linkages [10]. Insulin is released with an increase in blood glucose levels. It serves as the primary glucose concentration regulator, which promotes glucose uptake in fat and skeletal muscle and inhibits hepatic glucose production [11]. Insulin promotes substrate storage by stimulating glycogen synthesis, protein synthesis, and lipogenesis, while simultaneously inhibiting lipolysis, glucogenolysis, and protein breakdown [11,12]. Moreover, insulin regulates cell growth and differentiation, indicating the profound roles of insulin in overall cell physiology. The resistance and dysregulation of insulin signaling induces the elevation of fasting and postprandial glucose, as well as lipid levels. It also leads to the dysregulation of these processes [11]. mTOR is a master regulator of cell growth and is also closely implicated in metabolic changes in the liver, adipose tissue, and muscle upon postprandial elevation of insulin levels [2,13,14,15]. This review summarizes the major findings on the involvement of mTOR in insulin signaling, with particular emphasis on the molecular regulation of insulin signaling.

## 2. Regulation of mTORC1

### 2.1. Upstream and Downstream Targets of mTORC1

As a master regulator of cell growth, mTORC1 is activated by several growth factors and mitogen-dependent pathways [12]. mTORC1 is also responsive to intracellular and environmental stresses that are opposed to cell growth such as low levels of adenosine triphosphate (ATP) or oxygen and DNA damage [16]. The tuberous sclerosis complex 1/2 (TSC1/2) and the small guanine-5′-triphosphate (GTP) ase, a RAS homolog enriched in the brain (RHEB), serve as a major hub for transducing the upstream signal to mTOR [7]. TSC acts as a GTPase-activating protein (GAP) for RHEB [12]. While GTP-loaded RHEB activates mTORC1, TSC negatively regulates RHEB and mTORC1 signaling [8]. Insulin-stimulated protein kinase B (PKB, also known as Akt) activates mTORC1 signaling by phosphorylating and inhibiting TSC2, enabled by the dissociation of TSC2 from the lysosomal membrane, where a fraction of RHEB is localized [17].

The two best-known downstream targets of mTORC1 are ribosomal protein S6 kinase 1 (S6K1) and eukaryotic translation initiation factor 4E-binding protein 1 (4EBP1) [7]. Activated mTORC1 phosphorylates the hydrophobic motif of S6K1, thus sequentially activating S6 to induce ribosome biogenesis [8]. The phosphorylation of 4EBP1 at multiple sites by mTORC1 inhibits 4EBP1, leading to the translational initiation [8]. Insulin also directly activates mTORC1 kinase activity, followed by increasing the association of 4EBP1 and raptor in mTORC1 [18]. The major insulin-responsive form of mTORC1 is the dimeric mTORC1 complex, a structure containing two heterodimers of mTOR, raptor, and mLST8 [18]. This complex has been confirmed by both cryo-electromicroscopy (EM) and single molecule pull-down (SiMPull) [19,20]. Two of the upstream stimuli of mTORC1, namely phosphatidic acid and amino acids, will be discussed in the following sections.

### 2.2. Phosphatidic Acid: An Upstream Stimulus of mTORC1

The lipid second messenger phosphatidic acid (PA) has been identified as a critical mediator of mTOR signaling [21]. PA physically associates with the FKBP12-rapamycin-binding (FRB) domains of mTOR surrounding amino acids 2015 to 2114 in a highly specific manner, as confirmed by nuclear magnetic resonance (NMR) spectroscopy [22,23]. Among several enzymes in PA biogenesis, phospholipase D1 (PLD1), which hydrolyzes phosphatidylcholine to produce phosphatidic acid, is a critical regulator of mTOR signaling induced by mitogens and amino acids [21,24,25]. Recent reports have revealed the mechanism of how PLD1-generated PA activates mTOR kinase activity. PA specifically activates mTORC1 activity in vitro without affecting mTORC2 activity [26]. Although PA maintains the stability of mTORC1 and mTORC2 at the steady state [27], PLD1-generated PA displaces DEPTOR from mTORC1 [28]. PLD1-generated PA has at least one unsaturated fatty acid chain [29], which confers affinity to the FK506 binding protein (FKBP)-rapamycin binding (FRB) domain of mTOR, leading to the displacement of DEPTOR and subsequent mTORC1 activation in vivo and in vitro [28].

### 2.3. Amino Acids: An Upstream Stimulus of mTORC1

Amino acid availability regulates mTORC1 activity through the RAS-related GTP-binding protein (Rag) family of small GTPases [30], which is distinct from the growth factors. Whereas growth factors regulate mTORC1 kinase activity, amino acid availability induces mTORC1 localization to the lysosome, which is essential for mTORC1 activation [31]. Rags form heterodimers of either Rag A or Rag B with either Rag C or Rag D [30]. Amino acids convert Rags to active conformation (Rag A or Rag B is loaded with GTP and Rag C or Rag D with GDP) [8,30]. Amino acid availability and mTORC1 translocation by Rag family GTPases are prerequisites for mTORC1 activation on the outer surface of the lysosome, where RHEB resides [30]. Ragulator (also known as LAMTOR: Lysosomal Adaptor and Mitogen-activated protein kinase and mTOR activator) is a pentameric complex that is responsible for tethering the Rag proteins to the lysosomal surface. It also acts as a guanine nucleotide exchange factor (GEF) for Rag A or Rag B [32,33]. In addition, several cell-based biochemical studies demonstrated that GAPs as well as other regulatory proteins operate upstream of Rag GTPases [34,35,36,37,38,39].

Vps34, a class III phosphoinositide 3-kinase (PI3K), also acts as an amino acid mediator in mTOR signaling [40,41]. In the presence of amino acids, Vps34 produces phosphatidylinositol-3-phosphate (PtdIns(3)P) from phosphatidylinositol (PtdIns), which is followed by mTORC1 activation. The mechanisms by which Vps34 regulates mTORC1 in amino acid signaling have been suggested. Gulati et al. proposed that amino acids enhance the interaction of Ca^2+^/calmodulin (CaM) with Vps34-mTOR by facilitating Ca^2+^ influx, resulting in mTORC1 activation [42]. A recent study identified leucyl-tRNA synthetase (LeuRS) as an amino acid sensor of Vps34 [43]. In addition to the conservative LeuRS function of producing Leu-tRNA, LeuRS has been identified as a cytosolic amino acid sensor in mTORC1 signaling, serving as a GAP for Rag D [44]. Moreover, LeuRS binds to Vps34 via the minimum-binding site (amino acid 361 to 720) at the N-terminus and activates Vps34 through the C-terminal unique domain of LeuRS, UNE-L, in a nonautophagic complex (Atg14L-deficient Vps34 complexes), in which amino acids activate Vps34 kinase activity [43,45]. Vps34-generated PtdIns(3)P activates PLD1 through the Phox (PX) domain of PLD1 and translocates PLD1 to the lysosome where mTORC1 is recruited [25]. PLD1 translocation to the lysosome parallels Rag-regulated mTORC1 localization to the lysosome, although LeuRS acts as a cytosolic amino acid sensor in both processes [25]. Whether LeuRS prefers one of these parallel pathways in certain biological contexts requires further investigation.

## 3. Regulation of mTORC2

In contrast to mTORC1 regulation, mTORC2 regulation is poorly understood. Active mTORC2 phosphorylates the hydrophobic motif in a subset of AGC (cyclic adenosine monophosphate (cAMP)-dependent, cyclic guanosine monophosphate (cGMP)-dependent, and protein kinase C) family kinases, including Akt, serum/glucocorticoid-regulated kinase 1 (SGK1), and protein kinase C (PKC) [8]. Among the mTORC2 substrates, Akt regulates mTORC2 activity by forming a positive feedback loop [46]. 3-phosphoinositide-dependent protein kinase 1 (PDK1)-stimulated Akt phosphorylates SIN1 at threonine (T) 36, resulting in further mTORC2 activation to then fully activate Akt by phosphorylating serine (S) 473 [46].

mTORC2 is stimulated by growth factors such as insulin, insulin like growth factor (IGF), and hormones, which signal to PI3K [47]. Although both mTORC1 and mTORC2 are activated by growth factors, including insulin, the signaling mechanism of mTORC2 activation is distinct from that of mTORC1 [8]. PI3K dependent insulin signaling promotes the association of mTORC2 with ribosomes that increase mTORC2 activity [48]. In line with this, elevated PI3K signaling increases the binding of mTORC2 to ribosomes in cancer cells [49]. Ribosomes function as a scaffold for mTORC2 phosphorylation to substrates or confer the proper localization of mTORC2. However, the mechanism that induces mTORC2 activation needs to be elucidated [49].

Based on mTORC2 dependency on PI3K in insulin signaling, whether the mTORC1-driven negative feedback loop could affect mTORC2 activity is worth investigation. Using a data-driven dynamic insulin mTOR network model that integrates the entire core network, Pezze et al. suggested that mTORC2 activity is dependent on PI3K activity, which is insensitive to the mTORC1-dependent negative feedback loop [50]. This study also indicated that TSC complexes, a negative upstream regulator of mTORC1, are not required for insulin-induced mTORC2 activation [50]. However, the requirement for the TSC1-TSC2 complex was suggested for proper mTORC2 activation based on their physical interaction [51]. The existence of a negative feedback loop-independent of PI3K is conceivable and requires further investigation.

Upon growth factor stimulation, ras-related C3 botulinum toxin substrate 1 (Rac1) GTPases are proposed to translocate to the plasma membrane and recruit mTORC2 for the regulation of its activity [52]. In line with this observation, leucine induces Rac activation and cell migration through phosphatidylinositol 3,4,5-triphosphate-dependent Rac exchanger 1 (P-Rex1) and P-Rex2 in an mTORC2 dependent manner [53]. Additionally, P-Rex1 also associates with both mTORC1 and mTORC2, but activates only mTORC2 [53]. However, the mechanism of Rac1 and P-Rex-induced mTORC2 activation needs to be further clarified.

## 4. Insulin Signaling

Insulin binding to its receptor evokes a series of signaling events. Insulin receptor (IR) belongs to a subfamily of receptor tyrosine kinases that include the IGF-I receptor and the insulin receptor-related receptor (IRR) [11]. These receptors consist of two extracellular α-subunits (135 kDa) and two transmembrane β subunits (95 kDa), which form a tetrameric protein complex [54]. Insulin binding to its receptor leads to the autophosphorylation of its receptor on tyrosine residues of the β subunit (Tyr1158, Tyr1162, and Tyr1163), resulting in the activation of IR tyrosine kinase. The activated IR tyrosine kinase recruits its substrates and phosphorylates their tyrosine residues [55]. The family of insulin receptor substrates (IRSs: IRS-1, IRS-2, IRS-3, and IRS-4) and other substrates (GRB2-associated-binding protein1 (Gab-1), docking protein P62^dok^, E3 ubiquitin-protein ligase Cbl, adapter protein containing PH and SH2 domain (APS), and SHC-transforming protein (Shc)), mediating the binding of their effectors, which contain Src homology domain2 (SH2), unlike other receptor tyrosine kinases that directly bind their effectors [56,57].

While IR and IRS proteins are activated by tyrosine phosphorylation, they are inhibited by protein tyrosine phosphatases (PTPs) and serine phosphorylation [56]. Serine phosphorylation of IR and IRS proteins impairs insulin stimulated signaling by reducing their tyrosine phosphorylation and promoting their interaction with 14-3-3 [11,58]. The rapid dephosphorylation of tyrosine phosphorylation by PTPs also attenuates insulin signaling. Knockout of protein tyrosine phosphatase 1B (PTP1B), a cytoplasmic PTP, increased tyrosine phosphorylation of IR and IRS-1 in muscle, leading to an increase in insulin sensitivity [59,60].

The association of IRSs with p85, the regulatory subunit of PI3K [61], leads to the recruitment of p110, a PI3K catalytic subunit. p110 is recruited near its physiological substrate phosphatidylinositol (4,5) bisphosphate (PtdIns(4,5)P2), which it phosphorylates at the D3 position of the inositol ring to generate PtdIns(3,4,5)P3 [62]. The subsequent increase of PtdIns(3,4,5)P3 leads to the recruitment of its key effector of insulin signaling, Akt from the cytosol to the plasma membrane by binding to a pleckstrin homology (PH) domain in the amino terminus of Akt [62]. This association brings Akt close to PDK1 and mTORC2 (functions as PDK2), leading to the phosphorylation of Akt on threonine 308 and serine 473, respectively [8].

Activated Akt is dissociated from the plasma membrane and is phosphorylated by a myriad of substrates, which is important for regulating insulin dependent processes [62]. Glycogen synthase kinase 3 (GSK-3) is inactivated following its phosphorylation by Akt [11], leading to the activation of glycogen synthase, which catalyzes the final step of glycogen synthesis [62]. Akt also phosphorylates and inhibits the Rab-GTPase-activating protein, AS160 (for Akt substrate of 160 KD) [63]. This prompts the translocation of the glucose transporter GLUT4 to the plasma membrane by activating Rab small GTPases and controlling cytoskeletal reorganization [63]. In addition, Akt phosphorylates several of the winged helix or forkhead box protein O (FOXO) class of transcription factors that are critical for the expression of gluconeogenic and lipogenic enzymes. For example, FOXO1 activates gluconeogenic genes in the liver [64] and inhibits adipogenesis [65]. Insulin-activated Akt phosphorylates FOXO1 at serine 256, facilitating the interaction with 14-3-3 proteins and sequestration into the cytoplasm, leading to the inhibition of the expression of gluconeogenic enzymes [56]. Akt also regulates mTORC1 signaling by phosphorylating and inhibiting TSC1/2, a negative regulator of mTORC1 [8].

Recently, the Hippo pathway has been shown to affect insulin signaling via its regulation of mTORC1 and mTORC2 [8]. The Hippo pathway plays a role in the determination of organ size by restraining the cell number through the enhancement of apoptosis and the suppression of cell proliferation [66]. G-protein coupled receptors (GPCRs) inhibit the Hippo pathway component, large tumor suppressor homolog (LATS) kinase, resulting in hypophosphorylation of the Yes-associated protein (YAP) that translocates to the nucleus [67,68]. YAP promotes the expression of the microRNA (miR)-29, which targets phosphatase and tensin homolog (PTEN) mRNA and inhibits PTEN translation [67]. The decrease in the level of PTEN, a negative regulator of PI3K-Akt signaling, increases the level of PtdIns(3,4,5)P3, leading to a further activation of both mTORC1 and mTORC2 in insulin signaling.

## 5. IRS Regulation by mTORC1

**Figure 2:**
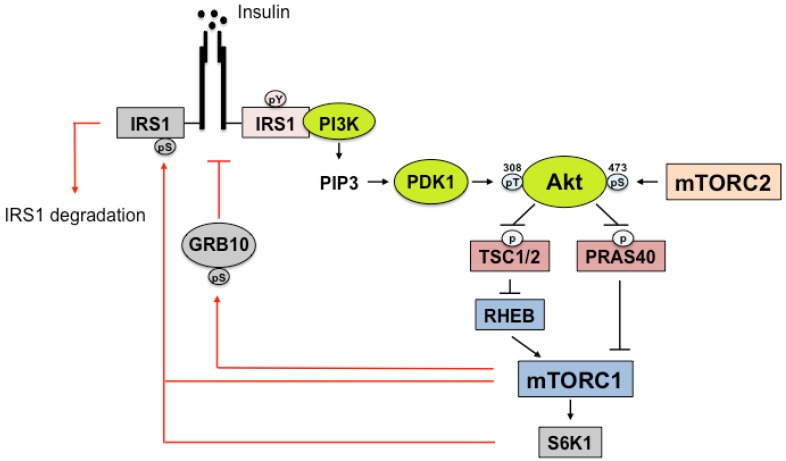
Negative feedback regulation of insulin receptor substrate (IRS) by rapamycin complex 1 (mTORC1) Activated mTORC1/S6K1 inhibits IRS-1 by phosphorylating serine residues. mTOR phosphorylates mouse (m)S632 (human (h)S636), mS307 (hS312), and mS612 (hS616), and S6K1 phosphorylates mS302 (hS307), mS522 (hS527), mS265 (hS270), and mS1097 (hS1101). mTORC1 phosphorylates and stabilizes Grb10, which inhibits the interaction between IRS and the phosphorylated tyrosine of IR. Akt indirectly activates mTORC1 by phosphorylating tuberous sclerosis complex (TSC) and PRAS40. The gray images are mTORC1 targets in insulin signaling. The red line represents negative regulation. S6K1, ribosomal protein S6 kinase 1; pY, phosphotyrosine; pS, phosphoserine; PI3K, phosphoinositide 3-kinase; PIP3, phosphatidylinositol (3,4,5)-triphosphate; PDK1, 3-phosphoinositide-dependent protein kinase 1; pT, phosphothreonine; Akt, protein kinase B (PKB); GRB10, growth factor receptor-bound protein 10; PRAS40, proline-rich AKT substrate of 40 kDa; RHEB, RAS homolog enriched in the brain.

IRS-1 and IRS-2 contain highly similar amino-terminal pleckstrin homology (PH) and phosphotyrosine-binding (PTB) domains (~100 amino acids each), followed by long C-terminal tail regions that are apparently unstructured, whereas only IRS-2 includes the kinase regulatory loop-binding (KRLB) domain [58,69]. In addition, IRS interacts with a key phosphotyrosine in IR, which is inhibited by growth factor receptor-bound protein 10 (Grb10). mTORC1 phosphorylates and stabilizes Grb10 to block insulin signaling [70] (Figure 2).

Insulin-IRS-induced Akt activation results in indirect mTORC1 activation through the phosphorylation of TSC and PRAS40. Akt directly phosphorylates TSC2 at two sites (S939 and T1462 on the full length of the human protein) and possibly at two or three more sites (S981 and S1130/S1132) [71]. TSC2 forms a complex with TSC1 and functions as a GAP for RHEB, as discussed in Section 2. Additionally, Akt directly phosphorylates PRAS40 at T246 [72]. PRAS40 has been shown to interact with mTORC1, which negatively regulates mTORC1 signaling [73]. Phosphorylated PRAS40 binds to 14-3-3 proteins, which have been proposed to sequester PRAS40 away from mTORC1 [74]. In addition to Akt signaling to mTORC1, nutrient signals, (e.g., amino acids, especially branched chain amino acids (BCAAs)) stimulate mTORC1/S6K1 independently of Akt-IRS-1.

Serine/threonine phosphorylation of IRS affects its tyrosine dephosphorylation, its dissociation from IR, its intracellular localization, and its eventual degradation in a proteasome dependent manner through the different mechanisms [58,75,76]. Activated mTORC1 negatively regulates insulin signaling by phosphorylating IRS-1 at serine residues. In vitro studies demonstrated that S6K1 phosphorylates mouse (m)S302 and mS522 (human (h)S307 and hS527) of IRS-1 [77], and mTORC1 phosphorylates mS632 (hS636) of IRS-1 [78]. In addition, in vivo S6K1 phosphorylates other S/T residues (mS265/hS270 and mS1097/hS1101) [79,80], whereas mTORC1 and other kinases phosphorylate S/T residues (mS307 /hS312 and mS612/hS616) [77]. Genetic deletion of S6K1 protects mice from obesity and insulin resistance under high fat diet (HFD)-fed conditions. In these mice, IRS-1 phosphorylation at S302, S632 (in adipose tissue), and S1097 (in liver) are reduced to similar levels in regular chow (RC)-fed mice, supporting the role of active S6K1 in IRS-1 phosphorylation at serine residues [13]. However, raptor binds to Shc and IRS-1 NPXY binding (SAIN) domain of IRS-1 and phosphorylates S636/639 by mTOR [78]. This is regulated in a nutrient dependent and S6K1 independent manner. These results suggest that mTORC1 and S6K1 regulate IRS-1 independently. A recent study using quantitative mass-spectrometry of hyperinsulinemic-euglycemic clamped human muscle demonstrated that the desensitization of insulin signaling correlates with the phosphorylation of IRS-1 at hS312, hS616, hS636, and hS1101, supporting the negative feedback regulation of mTORC1-S6K1 [81]. The over-supply of nutrients in obesity is suspected to activate mTORC1, resulting in an increase of IRS phosphorylation at serine residues and the development of insulin resistance. Therefore, IRS acts as not only an upstream regulator of mTORC1 but also as a receiver of feedback regulation through Ser/Thr phosphorylation (Figure 2).

However, several reports have suggested that serine phosphorylation correlates with insulin sensitivity. hS307 (mS302) phosphorylation of IRS-1 promotes insulin signaling in cell culture, which affects the insulin-stimulated tyrosine phosphorylation of IRS-1 and p85 binding, as well as mTORC1 signaling [82]. Consistent with this study, hS307 phosphorylation of IRS-1 in human primary adipocytes correlates with the insulin sensitivity in an mTORC1 dependent manner [83] and is attenuated in adipocytes of patients with type 2 diabetes [84]. IRS-1 mS307 (hS312) has been shown to protect mice against HFD-induced insulin resistance without affecting phosphorylation at other serine residues and while maintaining PI3K binding to IRS-1 [85]. Furthermore, several studies suggest that serine phosphorylation of IRS-1 serves as a priming signal to insulin signaling [86] or reduces tyrosine dephosphorylation [87] and inhibitory S/T phosphorylation [88] to strengthen the output insulin signal. The mechanism by which mTORC1/S6K1-induces IRS-1 serine phosphorylation in opposite directions of insulin signaling warrants further investigation.

## 6. IR/IRS Regulation by mTORC2

As discussed above, the roles of mTORC1 in activating and down regulating insulin signaling have been relatively well characterized. On the other hand, the positive role of mTORC2 in IGF signaling was suggested through its regulation of IGF II translation [89]. mTORC2 promotes IGF II translation by phosphorylating the RNA-binding protein IMP1 (IGF2 mRNA-binding protein 1) at serine 181. S181 phosphorylation of IMP1 strongly increased the binding of IMP1 to the 5′ untranslated region of IGF II-leader 3, which is essential for its mRNA translational initiation via internal ribosomal entry.

Recently, Yin et al. demonstrated the direct regulation of mTORC2 in IGF-IR and IR [90]. Unexpectedly, in addition to the well-established mTOR serine/threonine kinase activity, mTOR directly phosphorylates tyrosine residues in IGF-IR/IR in a cell free system [90]. Rictor is required for the tyrosine kinase activity of mTOR in cells. In addition, IRS-1 directly interacts with SIN1 to recruit mTORC2 to IGF-IR/IR and promote the phosphorylation of IGF-IR/IR on Y1131/1136 and Y1146/1151. These phosphorylations are not regulated by the intrinsic kinase activity of IGF-IR/IR. Hence, these results suggest that mTORC2 functions as a tyrosine kinase for IGF-IR/IR.

In addition, the role of mTORC2 was suggested in IRS-1 degradation. Kim et al. reported that mTORC2 negatively regulates IRS-1 levels by regulating the stability and localization of F-box/WD repeat-containing protein 8 (Fbw8), the substrate-targeting subunit of the cullin-7 (CUL7) E3 ligase complex [91]. mTORC2 phosphorylates Fbw8 to enable its translocation to the cytosol upon insulin stimulation. Fbw8 mediates the ubiquitylation and degradation of IRS-1, which is required for the proper turnover of IRS-1 and the removal of inactive IRS-1 in the cytosol [91]. However, other reports showed discrepant observations that acute rapamycin treatment or mTORC2 inhibition did not decrease IRS-1 [78,92], which is requires further investigation.

## 7. The Role of mTOR in Insulin-Induced Glucose and Lipid Metabolism

**Figure 3:**
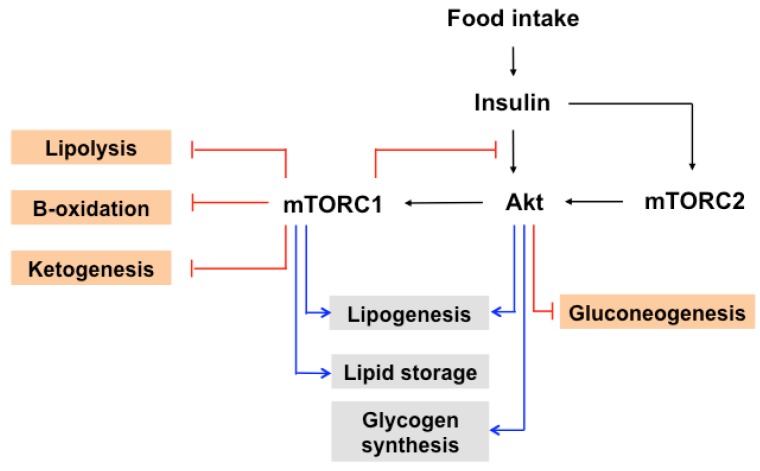
The role of mTOR in insulin induced metabolic processes. The postprandial increase of insulin activates protein kinase B (PKB, also known as Akt) through PDK1and mTORC2. Akt activates mTORC1 by phosphorylating TSC1/2. Activated mTORC1 phosphorylates IRS-1, leading to negative feedback regulation of block insulin signaling. mTORC1 and mTORC2 promote lipogenesis by regulating SREBP. mTORC1 enhances lipid storage, whereas it inhibits lipolysis, β-oxidation, and ketogenesis. In addition, mTORC2 promotes glycogen synthesis and decreases gluconeogenesis. The blue line represents positive regulation, and the red line represents negative regulation. The detailed regulation is indicated in the text. PDK-1; 3-phosphoinositide-dependent protein kinase 1, SREBP; sterol regulatory element-binding protein.

The postprandial increase of glucose and insulin activates mTOR within metabolic tissues to control whole body metabolic homeostasis (Figure 3). mTORC2 regulates glucose homeostasis through Akt. Akt promotes glucose uptake by increasing GLUT4 translocation to the membrane in adipocytes [93]. In addition, Akt phosphorylates and deactivates GSK-3, which decreases the rate of phosphorylation of glycogen synthase. This leads to an increase in glycogen synthase activity, thus elevating the accumulation of glycogen, which is especially important in the muscles and liver [71]. Akt also controls glucose homeostasis by phosphorylating and inhibiting FOXO1, a transcription factor that regulates gluconeogenesis [94].

mTOR signaling promotes hepatic lipogenesis through the regulation of sterol regulatory element-binding protein (SREBP)s, which include three isoforms, SREBP1a, SREBP1c, and SREBP2. Insulin-stimulated mTORC1/S6K1 facilitates to accumulate the mature form of SREBP1, activates the expression of SREBP1 and genes involved in both steroid and fatty acid biosynthesis, and elevates lipogenesis [95,96]. In addition, S6K1 promotes the processing of SREBP1 in the liver and of SREBP2 in a hepatocellular carcinoma cell line [97,98,99]. Additionally, mTORC1 phosphorylates and blocks the nuclear entry of the phosphatidic acid phosphatase lipin1, which has an inhibitory effect on nuclear SREBP levels by controlling nuclear lamina [100]. Further, mTORC2 plays an essential role in regulating SREBPs in mTORC1 dependent and independent manners [101]. Akt activates SREBP via the repression of the SREBP inhibitor Insig, Insig2a [102], or the inactivation of GSK3, which mediates SREBP phosphorylation and degradation [103].

mTORC1 is involved in promoting the storage of fatty acids in lipid stores by inhibiting lipolysis [2]. Elevated lipolysis was observed under the inhibition of mTORC1 activity in adipose tissue through genetic modulation (S6K1 knockout mice or 4EBP1/2 double knockout) or rapamycin treatment [13,104,105,106]. In addition, mTORC1 has been shown to have an impact on three lipases: adipose triglyceride lipase (ATGL), hormone-sensitive lipase (HSL), and lipoprotein lipase (LPL) [2]. ATGL in adipocytes catalyzes the lipolysis of triacylglycerol (TAGs) to diacylglycerol (DAG), and then HSL converts DAG to monoacylglycerols (MAGs). mTORC1 suppresses the transcription of ATGL [14], reduces HSL activity by phosphorylating at Ser563 [106], and decreases the activity of the extracellular lipase LPL [107], a water-soluble lipase in plasma that facilitates the uptake of lipoprotein in tissues.

In line with the negative role of mTOR in lipolysis, mTORC1 is implicated in the inhibition of the β-oxidation of fatty acids and ketogenesis. mTORC1 enhances the nuclear accumulation of nuclear receptor corepressor 1 (NcoR1) and thus inhibits the activity of PPARα, resulting in the suppression of ketogenic gene expression [108]. mTORC1 decreases the β-oxidation of fatty acids and promotes mitochondrial biogenesis in some cellular contexts [2]. In addition, mTORC1 coordinates various levels of gene expression, leading to its control of the mitochondrial mass and functions. mTORC1 controls the 4EBP1-mediated translation of nuclear-encoded mitochondrial mRNAs such as transcription factor A, mitochondrial (TFAM), mitochondrial ribosomal proteins, and components of complex I and V [109] The inhibition of mTORC1 activity blocks the interaction of Yin-Yang 1 (YY1) with peroxisome proliferator-activated receptor gamma coactivator 1 (PGC1)-α, subsequently decreasing the expression of mitochondrial genes [110]. mTOR directly governs the transcription of Estrogen related receptor α (ERRα)-target genes that is responsible for mitochondrial function and energy metabolism [111].

As the central controller of nutrient-sensing signaling pathways and cell growth, mTOR is involved in β-cell survival and insulin secretion. The survival and death of β cells are affected by intracellular and extracellular nutrients such as glucose and amino acids [112,113]. β-cell mass is reduced in chronic rapamycin-treated rats [114]. S6K1 knockout mice have small-sized β cells [13]. Mice with rictor deficiencies exhibit mild hyperglycemia and glucose intolerance, owing to reduced β-cell mass and defects in glucose-induced insulin secretion [115]. Hence, these observations suggest a crucial role of mTOR signaling in the growth and functions of β cells. Recently, mTOR has been shown to reduce the expression of thioredoxin-interacting protein (TXNIP), a potent inducer of β-cell death and oxidative stress, through its association with the carbohydrate-response element-binding protein (ChREBP)-Max-like protein (Mlx) complex [116]. mTOR deficiency reduces β-cell survival and aggravates oxidative stress, together with the elevation in TXNIP and ChREBP levels, suggesting the importance of the mTOR-controlled transcriptional regulation network in β-cell survival and glucose homeostasis [116]. However, Alejandro et al. showed that mTOR-induced signaling is required for β-cell functions, not for β-cell mass, under both normal and HFD conditions, using tetracycline-off inducible mice overexpressing a kinase-dead mTOR mutant in β cells [117]. mTOR activity is closely associated with the expression of Pdx-1, a critical transcription factor regulating pancreatic development and the function and survival of β cells [117]. Additional studies need to further dissect the role of mTOR in the survival and function of β cells.

## 8. mTOR and Autophagy

While mTOR promotes anabolic processes in response to growth factors and nutrients, it also inhibits catabolic processes, mainly autophagy, to support cell growth. Autophagy is a self-cannibalization that contributes to the removal of damaged cell components and provides the energy sources and substrates for protein synthesis under starvation [118]. A series of ATG proteins induces autophagosome formation by sensing stress and starvation. ULK1 (yeast Atg1 mammalian homolog) is a critical initiator of autophagosome formation, which is tightly regulated by mTORC1 and 5′-AMP-activated protein kinase (AMPK) [119]. Under the abundance of nutrients, mTORC1 binds to ULK1, phosphorylates ULK1 and the mammalian homolog of Atg13, and inhibits ULK1 activity, resulting in the overall inhibition of autophagosome formation [119]. When cells are starved, inactivated mTORC1 dissociates from the ULK1 complex (ATG101, Atg13, and FIP2000), freeing itself to initiate the autophagosome formation [118]. Of note, the dual regulation of ULK1 by AMPK and mTORC1 is important for determining the extent of autophagy induction [16]; AMPK directly phosphorylates ULK1 at Ser 317 and 377 and activates ULK1 under glucose starvation, whereas mTORC1 phosphorylates ULK1 at Ser 757 and dissociates ULK1-AMPK complex, inhibiting ULK1 with glucose [120]. In addition, mTOR directly phosphorylates ATG14 at multiple sites, which leads to the inhibition of Vps34 kinase activity and the subsequent production of PtdIns(3)P [121].

Moreover, mTOR controls autophagy at the transcriptional level; mTOR phosphorylates the transcription factor EB (TFEB) at Ser 211 and subsequently inhibits the nuclear translocation of TFEB [122,123]. TFEB belongs to the basic helix-loop-helix leucine-zipper family of transcription factors (TFs) and promotes the expression of lysosomal biogenesis and autophagic genes [124]. The nuclear localization of TFEB and its activity correlate to its phosphorylation; phosphorylated TFEB prefers to associate with the members of 14-3-3 tyrosine 3-monooxygenase/tryptophan 5-monooxygenase activation protein (YWHA) family and to remain in the cytosol [124].

Autophagy plays a cytoprotective role by maintaining the integrity of the endoplasmic reticulum (ER) and mitochondria [125,126]. Autophagy eliminates ubiquitinated proteins and damaged organelles, which could form toxic aggregates [127]. Mitochondrial dysfunction and damaged mitochondria result in the accumulation of reactive oxygen species (ROS), possibly leading to insulin resistance [127,128]. A recent study showed that the reverse of autophagic dysfunction enhances insulin sensitivity in the adipose tissue of obese mice [129], suggesting the correlation between autophagy and insulin sensitivity. Furthermore, the regulation of autophagy in β cells is important for the proper functioning of the ER and mitochondria, which is critical for β cell survival [130]. Interferon α-induced ER stress in β cells impairs insulin secretion [131]. β cell-specific ATG7 deletion reduces β-cell mass and pancreatic insulin levels, together with mitochondrial swelling and ER distension [132]. Additionally, autophagy is required for the homeostasis of glucose tolerance and β-cell hyperplasia under high-fat diet conditions [133]. Thus, autophagy can protect β cells from cellular damage and maintain β-cell functions regarding insulin sensitivity.

## 9. Conclusions and Future Directions

We have summarized and highlighted the studies of mTOR regulation in insulin signaling. mTORC1 and mTORC2 are the main protein kinases that phosphorylate and activate several downstream protein kinases and AGC family member kinases. mTOR senses the external and the internal signals, especially nutrient signals, to regulate cellular growth and survival. Insulin serves as a major controller of blood glucose levels to regulate the glucose uptake into muscle and fat and gluconeogenesis in the liver. Insulin also facilitates the accumulation of glycogen and lipid by promoting lipogenesis and glycogen and protein synthesis in the muscles, liver, and fat. A postprandial increase of insulin and glucose acutely activates mTOR within metabolic tissues, in which mTOR plays an important role in glucose and lipid metabolism.

Hyperactive mTORC1 has been observed in obesity and nutrient overload, probably due to hyperglycemia and hyperinsulinemia. The increased levels of BCAAs, mTORC1 stimuli, are closely connected to insulin resistance and obesity [134,135]. Chronic mTORC1 activation could further increase lipid storage in adipose tissues and develop insulin resistance via negative feedback regulation through the phosphorylation of IRS-1 at serine residues [7,134]. Thus it has been suggested that mTOR inhibitors might offer therapeutic benefits in metabolic diseases such as insulin resistance and obesity [2]. However, since mTOR inhibitors block mTORC2/Akt activity and thereby further exacerbate insulin insensitivity, it might be more promising to target mTORC1 specifically in a direct or indirect manner. There are two mTORC1–specific regulations, amino acid- or AMPK-dependent. Amino acids activate mTORC1 without affecting mTORC2 activity through amino acid sensors and mediators [16]. AMPK inhibits mTORC1 through the phosphorylation of the TSC-TBC complex and raptor [136]. Therefore, inhibitors of amino acid-mediated signaling or AMPK might provide beneficial metabolic effects; however, this requires further investigation.
